# Robust meta-analysis for large-scale genomic experiments based on an empirical approach

**DOI:** 10.1186/s12874-022-01530-y

**Published:** 2022-02-10

**Authors:** Sinjini Sikdar

**Affiliations:** Department of Mathematics and Statistics, Old Dominion University, Norfolk, VA USA

**Keywords:** Meta-analysis, Fisher’s *p*-value combination, Empirical null distribution, Weighted Z statistic, Simultaneous hypothesis testing

## Abstract

**Background:**

Recent high-throughput technologies have opened avenues for simultaneous analyses of thousands of genes. With the availability of a multitude of public databases, one can easily access multiple genomic study results where each study comprises of significance testing results of thousands of genes. Researchers currently tend to combine this genomic information from these multiple studies in the form of a meta-analysis. As the number of genes involved is very large, the classical meta-analysis approaches need to be updated to acknowledge this large-scale aspect of the data.

**Methods:**

In this article, we discuss how application of standard theoretical null distributional assumptions of the classical meta-analysis methods, such as Fisher’s *p*-value combination and Stouffer’s Z, can lead to incorrect significant testing results, and we propose a robust meta-analysis method that empirically modifies the individual test statistics and *p*-values before combining them.

**Results:**

Our proposed meta-analysis method performs best in significance testing among several meta-analysis approaches, especially in presence of hidden confounders, as shown through a wide variety of simulation studies and real genomic data analysis.

**Conclusion:**

The proposed meta-analysis method produces superior meta-analysis results compared to the standard *p*-value combination approaches for large-scale simultaneous testing in genomic experiments. This is particularly useful in studies with large number of genes where the standard meta-analysis approaches can result in gross false discoveries due to the presence of unobserved confounding variables.

**Supplementary Information:**

The online version contains supplementary material available at 10.1186/s12874-022-01530-y.

## Background

In genomic experiments and association studies, meta-analysis is a popular tool for pooling results from multiple experiments and research studies to reach an overall decision. Due to the rapid progress in technology, there has been major development of high-throughput genomic assays. It is now possible to analyze hundreds or thousands of genes at the same time. Thus, the paradigm of simultaneous inference has transformed a lot over the past few years. Moreover, huge number of available datasets in public repositories and databases have enabled researchers to assimilate large-scale genomic information from multiple studies in the form of meta-analysis [1–3]. Since the sample sizes of individual genomic experiments are generally small compared to the number of genes resulting in loss of power of statistical detection after adjusting for multiple testing, meta-analysis of multiple genomic experiments has been recognized as the appropriate method in order to achieve adequate sample sizes and optimal power for statistical detection [4, 5]. Meta-analysis has also gained popularity as a powerful tool for combining results from multiple genome-wide association studies [6, 7]. However, current meta-analysis approaches cannot accommodate the new large-scale aspect of the underlying inference of genomic experiments. The traditional meta-analysis methods, initially developed for combining results of significance testing from experiments involving only a few candidate genes, are still being applied to current large-scale experiments involving thousands of genes [8–10]. There are two main approaches for classical meta-analysis methods [11]. The first approach is to combine *p*-values of significance testing from multiple studies, and the second approach is to combine the model-based effect sizes from different studies. While both the approaches have their own advantages and disadvantages, the *p*-value combination methods are more flexible as they require less assumptions from the component studies and allows results from the component studies to be combined even when the individual effect sizes and standard errors are unavailable or are in different units. Some classical *p*-value combination methods include Fisher’s p-value combination [12], Stouffer’s Z-test [13], and the weighted variations of these methods [14]. In this paper we will focus on the meta-analysis methods of *p*-value combination.

One of the main assumptions of the classical *p*-value combination methods is that for a given gene, the *p*-values obtained from the component studies are individually uniformly distributed under the null hypothesis. However, as pointed out by Efron [15], in large-scale multiple testing problems, the *p*-values may not be uniformly distributed. Consequently, this raises questions on the validity of the distributional assumptions of *p*-value combined test statistics of the classical approaches of Fisher [12], Stouffer [13] and their variants. To apply these classical *p*-value combination approaches to large-scale significance testing, one needs to ensure that all the *p*-values obtained from the individual studies are uniformly distributed. This is important in such large-scale hypothesis testing frameworks since the aims of these experiments differ from that of a traditional single hypothesis testing scenario. In a single hypothesis test, one aims to reject an uninteresting null hypothesis in favor of some interesting alternative hypothesis with high power, e.g., 90%. However, in a large-scale genomic experiment, the number of hypotheses can easily be as large as 10,000 because of the same number of genes involved. In that case, the aim is to identify a small subset of genes, usually much less than 10% of the total number of genes, which are of most significance and are carried forward for further investigation. Thus, it is not expected from a large-scale multiple hypotheses framework to reject 90% of the 10,000 null hypotheses involved, unlike that of a single hypothesis framework. Efron [15] points out that the advantage of having thousands of null hypotheses in place of a single null hypothesis is that one can estimate the null distribution empirically and do not need to carry out testing based on some theoretical asymptotic null distribution. Empirical null distribution can be very useful in large observational studies since, unlike the theoretical null, it can take into account the additional variation and moderate bias caused by some unobserved variables (e.g., batch effects [16] or unmeasured confounder effects). Moreover, the problems caused by ignoring the effects of unobserved variables or potential confounders, and relying on theoretical null distribution for testing, can be aggravated in meta-analysis of large-scale genomic studies as discussed in Sikdar et al. [17]. In such a situation, even though a meta-analysis method has high power, it can lead to gross false discoveries of significant genes even after applying standard multiple testing correction techniques [17]. Therefore, in order to reduce the false discovery rate, it is essential to build meta-analysis methods involving large-scale hypothesis testing that are based on empirically adjusted null distribution rather than a theoretically assumed null distribution.

The idea of drawing inference based on an empirical null distribution, instead of a theoretical null distribution, was recently adopted in the context of meta-analysis by Sikdar et al. [17] and later applied in You et al. [18]. The meta-analysis method of Sikdar et al. [17], known as EAMA, modifies the classical Fisher’s *p*-value combination method for large-scale genomic studies by empirically adjusting the null distribution through an empirical Bayes framework where the amount of adjustment depends on the extent of discrepancy between the empirical and the theoretical null distributions. However, EAMA was only limited to the classical unweighted (equally weighted) Fisher’s *p*-value combination method which can perform poorly when there are large variations among the component studies of meta-analysis. Moreover, its performance can become unstable in certain situations as shown through simulation studies in a later section. In this article we propose a meta-analysis method for large-scale genomic experiments that implements a weighted *p*-value combination approach while estimating the empirical null distribution parameters through a recently developed Bayesian approach by van Iterson et al. [19] as opposed to the empirical Bayes approach of EAMA. Through a variety of simulated scenarios, we show that our proposed empirical null adjusted meta-analysis method has robust performances and works best in reducing false discoveries among several competing approaches for large-scale genomic meta-analysis especially in the presence of hidden confounders. Moreover, we demonstrate the utility of the proposed meta-analysis approach through a meta-analysis of lung cancer genomic studies.

The rest of the paper is organized as follows. In the Methods section, we describe the popular *p*-value combination approaches, methods for empirical null estimation, and our proposed combination of empirical null adjusted meta-analysis of large-scale simultaneous significance testing. In the Results section, we construct various simulation settings to compare the performances of our proposed meta-analysis approach with that of the other competing approaches. We also illustrate our approach through an application on a set of lung cancer genomic studies. The article ends with a discussion and a conclusion section.

## Methods

### Meta-analysis using weighted Z-scores

Suppose there are *K* independent studies and *G* genes in each study. Here the idea is to detect the genes that are related to the outcome of interest based on the results from the *K* independent studies. In other words, for each gene *j*, we want to test the overall null hypothesis *H*_*j*_: gene *j* does not contribute to the outcome of interest across all *K* independent studies, *j* = 1, 2, …, *G*. The general principle in the meta-analysis framework is to combine the results for each gene across the *K* independent studies to reach an overall decision for that gene.

In this section, we focus on a widely used weighted Z-score meta-analysis method based on *p*-values from independent studies [14], which is defined as follows: Suppose *N*_*i*_ denotes the sample size of study *i*, *i* = 1, 2, …, *K*. Let Δ_*ij*_ and *p*_*ij*_ denote the direction of effect and the *p*-value for gene *j* from study *i*, respectively, *i* = 1, 2, …, *K*; *j* = 1, 2, …, *G*. The weighted Z-score meta-analysis method converts the direction of effect and *p*-value observed in each study for each gene into a signed *Z*-score, which is defined as

$${Z}_{ij}={\Phi}^{-1}\left(1-\frac{p_{ij}}{2}\right)\times {\Delta }_{ij}\ \mathrm{for}\ \mathrm{gene}\ j\ \mathrm{in}\ \mathrm{study}\ i;i=1,2,\dots, K;j=1,2,\dots, G.$$

The signed *Z*-scores, for each gene, are then combined across studies in a weighted sum where the weights are proportional to the square-root of the sample size for each study [13, 14]. That is, for a gene *j*, the overall Z-score is defined as

$${Z}_j=\frac{\sum_{i=1}^K{Z}_{ij}{w}_i}{\sqrt{\sum_{i=1}^K{w}_i^2}}$$

where $${w}_i=\sqrt{N_i}$$; *i* = 1, 2, …, *K*, *j* = 1, 2, …, *G*.

Finally, an overall *p*-value for the gene *j* is obtained as *P*_*j*_ = 2(1 − Φ(|−*Z*_*j*_|)), *j* = 1, 2, …, *G*.

### Method for empirical estimation of null distribution

Suppose there are *G* null hypotheses (for example, corresponding to *G* genes) in a single study. Let the *p*-values corresponding to the null hypotheses be denoted as *p*_1_, *p*_2_, …, *p*_*G*_. Each *p*-value in the study can be converted into z-score as *z*_*j*_ = Φ^−1^( *p*_*j*_), *j* = 1, 2, …, *G*. Theoretically, the null distribution of *z*_*j*_ is *N*(0, 1), *j* = 1, 2, …, *G*.

However, the large-scale multiple testing situations enable us to estimate the null distribution of *z*_*j*_, *j* = 1, 2, …, *G*. In this section, we will discuss a Bayesian approach, named BACON [19], for estimating the null distribution empirically.

BACON assumes that the z-scores can be modeled by a three-component normal mixture, where one of the components represents the empirical null distribution and the other two components represent two separate non-null distributions. Here, the z-scores close to the central peak of the histogram are assumed to be generated from the null distribution, whereas those towards the left and right tails of the histogram are generated from the two different alternative distributions. The three-component normal mixture model is defined as follows:

$$f(z)=\sum_{k=1}^3{p}_k\phi \left(z,{\mu}_k,{\sigma}_k\right)$$

where $\sum_{k=1}^3{p}_k=1$ and *ϕ*(*z*, *μ*_*k*_, *σ*_*k*_) represent the density of *N*(*μ*_*k*_, *σ*_*k*_^2^), *k* = 1, 2, 3. This method uses a Gibbs sampling scheme to estimate the parameters of the mixture distribution, assuming conjugate prior distributions for the parameters as given below:

${\mu}_k \mid {\sigma_k}^2\sim N\left({\lambda}_k,\frac{{\sigma_k}^2}{\tau_k}\right)$; *σ*_*k*_^2^~*InverseGamma*(*α*_*k*_, *β*_*k*_) and (*p*_1_, *p*_2_, *p*_3_)~*Dirichlet*(*γ*_1_, *γ*_2_, *γ*_3_), *k* = 1, 2, 3.

We considered the same hyper-priors as suggested by van Iterson et al. [19]. The initial values are considered based on the median and median absolute deviation of the test statistics [19].

At each iteration the Gibbs sampling algorithm comprises of the following steps given that we have *G* genes resulting in *G* z-scores of the form *z*_*j*_ (*j* = 1, 2, …, *G*) and the associated outcome values *y*_*j*_ (*j* = 1, 2, …, *G*):

1. The unobserved data is generated as: ${x}_{jk}\sim Multinomial\left({\overset{\sim }{\pi}}_{jk}\right)$ where *π*_*jk*_ = *p*_*k*_*ϕ*(*z*_*j*_, *μ*_*k*_, *σ*_*k*_) and ${\overset{\sim }{\pi}}_{jk}$ is the normalized proportion so that $\sum_{k=1}^3{\overset{\sim }{\pi}}_{jk}=1$.
2. The following quantities are calculated: ${\eta}_k=\sum_{j=1}^G{1}_{\left({x}_{jk}\ne 0\right)}$, ${s}_k=\sum_{j=1}^G{y}_j{1}_{\left({x}_{jk}\ne 0\right)}$, and ${s}_k^2=\sum_{j=1}^G{y}_j^2{1}_{\left({x}_{jk}\ne 0\right)}$.
3. Samples are generated from the posterior distributions as follows:
  $${\displaystyle \begin{array}{c}{p}_k\sim Dirichlet\left({\gamma}_k+{\eta}_k\right);{\mu}_k \mid {\sigma_k}^2\sim N\left(\frac{\lambda_k{\tau}_k+{s}_k}{\eta_k+{\tau}_k},\frac{{\sigma_k}^2+{s}_k}{\eta_k+{\tau}_k}\right);\\ {}\frac{1}{{\sigma_k}^2} \sim \Gamma \left({\alpha}_k+\frac{1}{2}\left({\eta}_k+1\right),\frac{1}{\left({\beta}_k+\frac{1}{2}{\tau}_k{\left({\mu}_k-{\lambda}_k\right)}^2+\frac{1}{2}{s}_k^2\right)}\right)\end{array}}$$

A total of 5000 iterations and a burn-in period of 2000 iterations are recommended.

### Proposed meta-analysis method based on empirically modified weighted Z-scores

In this section, we describe our proposed approach of an empirically adjusted meta-analysis that combines appropriately weighted modified z-values and computes multiple testing corrected *p*-values where the modification involves transforming the raw z-values through an empirical correction of the null distribution. Following are the detailed steps of our proposed meta-analysis method.

Considering *K* independent studies and *G* genes in each study, let *z*_*ij*_ denotes the signed z-score, obtained through transformation from *p*-value *p*_*ij*_ and direction of the effect estimates ∆_*ij*_ as ${z}_{ij}={\Phi}^{-1}\left(1-\frac{p_{ij}}{2}\right)\times {\Delta }_{ij}$, *i* = 1, 2, …, *K*; *j* = 1, 2, …, *G*, as defined in the Methods section. Since, these z-scores *z*_*ij*_ may not follow *N*(0, 1) under the null hypotheses, we empirically estimate the parameters of the null distribution of the z-scores using BACON. Let ${\hat{f}}_0\left({\hat{\mu}}_B,{\hat{\sigma}}_B^2\right)$ denote the BACON estimated null distribution of the z-scores. Using the estimated null density, we define ${\tilde{z}}_{ij}=\frac{z_{ij}-{\hat{\mu}}_B}{{\hat{\sigma}}_B}$ as the modified z-score for gene *j* from study *i*, *i* = 1, 2, …, *K*; *j* = 1, 2, …, *G*. The modified z-scores ${\tilde{z}}_{ij}$ s, expected to be standard normally distributed under the null hypotheses, are then meta-analyzed using the weighted Z-score method as follows:

$${Z}_j=\frac{\sum_{i=1}^K{\tilde{z}}_{ij}{w}_i}{\sqrt{\sum_{i=1}^K{w}_i^2}}$$

for *j* = 1, 2, …, *G*; where ${w}_i=\sqrt{N_i}$, and *N*_*i*_ is the sample size of the study *i*; *i* = 1, 2, …, *K*. The overall *p*-value for gene *j* is obtained as *P*_*j*_ = 2(1 − Φ(|−*Z*_*j*_|)), *j* = 1, 2, …, *G*. The final *p*-values, *P*_*j*_ s, are corrected for multiple testing using the Benjamini-Hochberg (BH) method [20].

### Alternative choices for empirical null adjusted *p*-value combinations

In this section we discuss Fisher’s p-value combination, a popular alternative to the weighted Z-score combination, which directly combines the *p*-values instead of transforming them into z-values. In addition, we briefly discuss another potential choice for computing empirical null distribution through an empirical Bayes method that was first proposed by Efron [15] and subsequently adopted for meta-analysis in EAMA [17]. The reason for our discussion of these methods is that one can potentially combine any of the two *p*-value/z-value combination approaches with any of the two empirical null computation algorithms and each such combination leads to a different empirical adjusted meta-analysis. We compare the performances of each such combination to our proposed meta-analysis method in our simulations.

We briefly discuss the Fisher’s *p*-value combination method [12] and the empirical Bayes method for estimating null distribution [15] as follows.

#### Fisher’s *p*-value combination

Fisher’s method combines *p*-values across independent studies giving equal weights to all studies [12]. Assuming *K* independent studies and *G* genes in each study, for gene *j*, the test statistic for the Fisher’s method is defined as

$${F}_j=2{\sum}_{i=1}^K\left\{-\mathit{\log}\left({p}_{ij}\right)\right\},j=1,2,\dots, G$$

Under the null hypothesis that gene *j* does not contribute to the outcome of interest, the test statistic *F*_*j*_ follows a *χ*^2^ distribution with 2*K* degrees of freedom, assuming that the *p*-values *p*_*ij*_*s* are independently uniformly distributed on the interval [0, 1] for each *j*; *i* = 1, 2, …, *K*; *j* = 1, 2, …, *G*.

#### Empirical Bayes method for estimating null distribution

Efron [15] used an empirical Bayes model for estimating the null distribution of the *z*-scores. The z-scores for the genes are classified into two classes – “uninteresting” if *z* is generated from the null distribution, and “interesting” if *z* is generated from the non-null distribution with respective densities *f*_0_(*z*) and *f*_1_(*z*). Also, let the prior probabilities of *z* belonging to the “uninteresting” or “interesting” classes be denoted as *p*_0_ and *p*_1_ = 1 − *p*_0_, respectively. The mixture density of the *z*-scores can be defined as *f*(*z*) = *p*_0_*f*_0_(*z*) + *p*_1_*f*_1_(*z*).

Following Bayes theorem, the a posteriori probability of belonging to the “uninteresting” class given *z* can be defined as

$$P\left(``\mathrm{uninteresting}"|\ z\right)=\frac{p_0{f}_0(z)}{f(z)}$$

The aim is to estimate the null density, *f*_0_, from the central peak of the histogram of the *z*-scores. Assuming the null density, *f*_0_ is *N*(*δ*_0_, *σ*_0_^2^), where the mean *δ*_0_ is not necessarily 0 and standard deviation *σ*_0_ is not necessarily 1, for all *z*-scores close to 0, we can write

$$\mathit{\log}\left(f(z)\right)=-\frac{1}{2}{\left(\frac{z-{\delta}_0}{\sigma_0}\right)}^2+ constant$$

The parameter *δ*_0_ can be estimated as *argmax*(*f*(*z*)) and *σ*_0_ can be estimated as ${\left[-\frac{d^2}{d{z}^2}\mathit{\log}\left(f(z)\right)\right]}_{{\hat{\delta}}_0}^{-\frac{1}{2}}$. However, the estimate of *σ*_0_ obtained by directly differentiating the spline estimate of *log*(*f*(*z*)) can be unstable. Therefore, one more smoothing step is applied where a quadratic curve, ${a}_0+{a}_1{x}_k+{a}_2{x}_k^2$ is fitted by ordinary least squares to the estimated *log*(*f*_*k*_) values, for *x*_*k*_ within 1.5 units of the maximum *δ*_0_, which yields ${\sigma}_0={\left[-2{a}_2\right]}^{-\frac{1}{2}}$. This approach of estimating the null distribution is called “central-matching” approach. More details about this approach can be found in Efron [15] and Efron [21]. This empirical Bayes method of estimating null distribution is referred to as EB method from now on.

Note that, incorporating EB adjustment into Fisher’s *p*-value combination approach leads to the previously mentioned EAMA method [17]. Following this approach, one can also apply EB adjustments to the weighted Z-scores method as well as BACON adjustments to Fisher’s *p*-value combination where each combination gives rise to a different meta-analysis method. The last two meta-analysis methods, namely, EB adjusted weighted Z-score and BACON adjusted Fisher, are also new and have not been explored before in the literature. In this article we are implementing them for the first time and will explore their performances as competing candidates to our proposed meta-analysis method in various simulation settings in the next section. We will also compare the performance of EAMA in that section.

## Results

### Simulation studies

To evaluate the performance of our proposed method, we simulated continuous gene expression datasets for multiple independent experiments. We considered three simulation settings – setting 1, setting 2, and setting 3. In setting 1 we assumed there exists no hidden variable or confounder in the data. For setting 2, we assumed presence of a hidden variable which acts as a confounder, and in setting 3 we assumed presence of a hidden variable which does not act as a confounder. Details of the data generation method are described below.

We considered 10 independent experiments, i.e. *K* = 10 and two groups of subjects. The total number of genes in each experiment was 10,000, i.e. *G* = 10,000, out of which 1000 genes were assumed to be differentially expressed between the two subject groups. The log expression value, *Y*_*jlm*_, for the gene *j*, subject *m* in group *l* was generated using a linear model as given below.

$${Y}_{jl m}=\mu +{\alpha}_j+{\beta}_l+{\left(\alpha \beta \right)}_{jl}+{\gamma}_{jl m}+{e}_{jl m};\kern0.5em j=1,2,\dots, G,l=1,2,m=1,2,\dots, {n}_l$$

where *n*_*l*_ denotes the number of subjects in each group ,*l* = 1, 2. Here, *μ* denotes the general mean effect, *α*_*j*_ denotes the effect due to the gene *j*, *β*_*l*_ denotes the effect due to the group *l*, (*αβ*)_*jl*_ denotes the interaction effect between the gene *j* and group *l*, *γ*_*jlm*_ denotes the effect of a hidden variable or confounder, which remains unaccounted during an analysis, on the gene *j*, subject *m* in the group *l*, while *e*_*jlm*_ denotes the error term.

For all simulations, we set *μ* = *α*_*j*_ = *β*_*l*_ = 0, for all *j*, *l* for simplicity. The interaction terms (*αβ*)_*jl*_ were generated as: For *j* ≤ 400, (*αβ*)_*j*1_ =  − 4, (*αβ*)_*j*2_ = 4; for 401 ≤ *j* ≤ 1000, (*αβ*)_*j*1_ = 4, (*αβ*)_*j*2_ =  − 4; and for *j* > 1000, (*αβ*)_*j*1_ = (*αβ*)_*j*2_ = 0. Generation of the interaction terms in this way ensures that only the first 1000 genes were differentially expressed between the two subject groups.

We considered four sets of correlated genes in each experiment as follows: *S*_1_ = {*j* : 1 ≤ *j* ≤ 1000}, *S*_2_ = {*j* : 4001 ≤ *j* ≤ 5000}, *S*_3_ = {*j* : 5001 ≤ *j* ≤ 5200}; and *S*_4_ = {*j* : 8091 ≤ *j* ≤ 9100} and $S=\bigcup_{u=1}^4{S}_u$. We generated correlated expression levels of the genes in the four clusters through the generation of the error terms, *e*_*jlm*_, as

$${\displaystyle \begin{array}{cc}{e}_{jlm}=\left\{\begin{array}{cc}\frac{1}{\sqrt{2}}{e}_{jlm}^{(1)}+\frac{1}{\sqrt{2}}{e}_{jlm}^{(2)}& if\ j\in S\\ {}{e}_{jlm}^{(2)}& otherwise\end{array}\right.& j=1,2,\dots, G,l=1,2,m=1,2,\dots, {n}_l\end{array}}$$

Here, ${e}_{jlm}^{(1)}$ were independently generated from *N*(0, 1). We considered the same value of ${e}_{jlm}^{(1)}$ for all the genes belonging to the same cluster. ${e}_{jlm}^{(2)}$ were generated independently from *N*(0, 2^2^); *j* = 1, 2, …, *G*, *l* = 1, 2, *m* = 1, 2, …, *n*_*l*_.

With the above choices of the parameters of the linear model, we generated datasets for the following three simulation settings:

- Setting 1: In this setting, we assumed that there does not exist any effect of hidden variable or confounder. So, *γ*_*jlm*_ = 0 for all *j*, *l*, *m*.
- Setting 2: In this setting, we assumed that there exists an effect of hidden variable which acts as a confounder. Here, we generated *γ*_*jlm*_ as *γ*_*jlm*_ = *u*_*jlm*_*I*(*s*_*jlm*_ = 1), where *s*_*jlm*_ were generated from *Bernoulli*(0.4) and *u*_*jlm*_ were generated depending on the gene, subject and also the experiment as follows:
  $${\displaystyle \begin{array}{cc}{u}_{j1m}\sim \left\{\begin{array}{cc}N\left(-1+i,{0.01}^2\right)& for\ j\le 400\\ {}N\left(2+i,{0.01}^2\right)& for\ 401\le j\le 1000\\ {}N\left(5+i,{0.01}^2\right)& for\ j>1000\end{array}\right.;& i=1,2,\dots, K;m=1,2,\dots, {n}_1\\ {}{u}_{j2m}\sim \left\{\begin{array}{cc}N\left(3+i,{0.01}^2\right)& for\ j\le 400\\ {}N\left(6+i,{0.01}^2\right)& for\ 401\le j\le 1000\\ {}N\left(9+i,{0.01}^2\right)& for\ j>1000\end{array}\right.;& i=1,2,\dots, K;m=1,2,\dots, {n}_2\end{array}}$$

Here, the effect of the hidden confounder varied between the two groups of subjects, according to the different groups of genes and over different experiments.

- Setting 3: In this setting, we assumed that there exists an effect of hidden variable which does not act as confounder. Therefore, we considered a simulation setting where the distribution of the hidden variable does not differ between the two subject groups. We generated *γ*_*jlm*_ as *γ*_*jlm*_ = *u*_*jlm*_*I*(*s*_*jlm*_ = 1), where *s*_*jlm*_ were generated from *Bernoulli*(0.4) distribution and *u*_*jlm*_ were generated as *u*_*jlm*_~*N*(5 + *i*, 0.01^2^); *i* = 1, 2, …, *K*; *m* = 1, 2, …, *n*_*l*_; *l* = 1, 2.

We considered different choices for the sample sizes of the experiments and the two groups in each experiment in our simulations which will be discussed in the later sections.

After generating the data for the three simulation settings in each experiment, we used the ‘limma’ package in Bioconductor for testing for differential expression for the genes between the two subject groups [22]. The raw *p*-value and direction of effect for each gene, obtained from ‘limma’, were stored. We applied our proposed method (BACON-adjusted weighted Z-score) to identify the significant set of genes. For comparison, we applied EB adjusted weighted Z-score method, EAMA, BACON-adjusted Fisher method, along with standalone Fisher’s method and weighted Z-score method without any empirical adjustments to identify the significant genes. A gene is identified as differentially expressed if the BH adjusted *p*-value is less than 0.05.

The performance of our proposed method and all the other methods in comparison were assessed using four measures, namely, sensitivity, specificity, false discovery rate (FDR) and false non-discovery rate (FNR) based on 500 independent Monte-Carlo iterations. We compared the performances of all the methods mentioned above under the following simulation scenarios for the three settings:

#### Unequal sample sizes of the two subject groups

When the number of samples in the two groups were unequal, we considered the total effective sample size for the experiment as $\frac{4}{\frac{1}{n_1}+\frac{1}{n_2}}$. In this simulation scenario, we considered *n*_1_ = 30 and *n*_2_ = 70 in each experiment. Therefore, the total effective sample size for experiment *i* is ${N}_i=\frac{4}{\frac{1}{n_1}+\frac{1}{n_2}}=84$, *i* = 1, 2, …, *K*. Table 1 shows the FDR values for our proposed method and all the other methods in comparison, for the three simulation settings.

**Table 1:** FDR of our proposed meta-analysis method (BACON adjusted Weighted Z) and the other methods in comparison with unequal sample sizes of the subject groups

Setting	Method	FDR
1	Fisher	0.05
	EAMA	0.05
	BACON adjusted Fisher	0.05
	weighted Z	0.05
	EB adjusted weighted Z	0.06
	BACON adjusted weighted Z	0.05
2	Fisher	0.83
	EAMA	0.06
	BACON adjusted Fisher	0.02
	weighted Z	0.90
	EB adjusted weighted Z	0.05
	BACON adjusted weighted Z	0.04
3	Fisher	0.05
	EAMA	0.11
	BACON adjusted Fisher	0.06
	weighted Z	0.05
	EB adjusted weighted Z	0.02
	BACON adjusted weighted Z	0.05

**Fig. 1:**
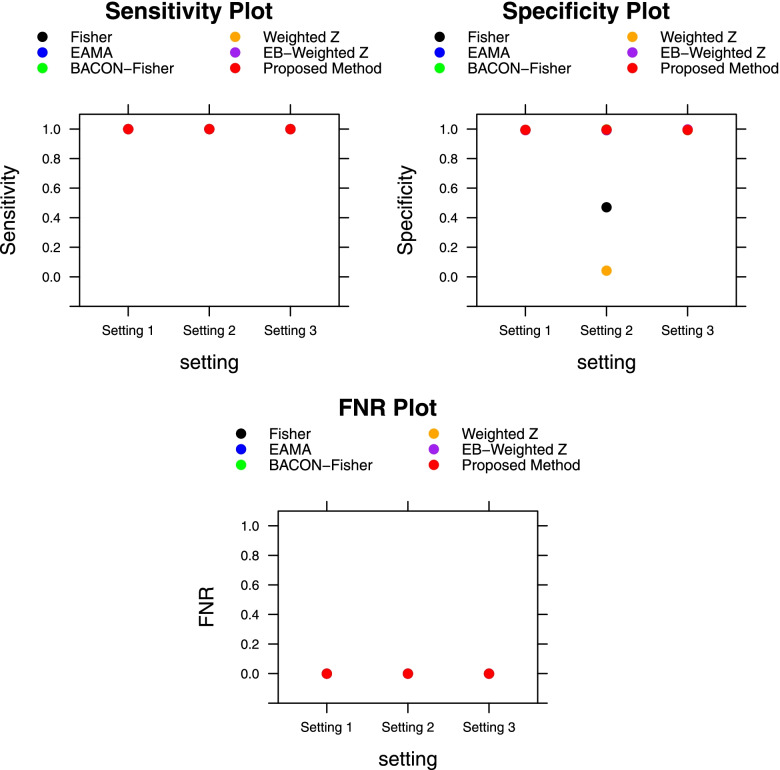
Performances of the meta-analysis methods in terms of sensitivity, specificity, and FNR with unequal sample sizes of the subject groups. This figure shows the average sensitivity, specificity, and FNR values over 500 independent Monte-Carlo iterations of the proposed method and all the other methods in comparison. Results are shown for all three simulation settings. The sample sizes of the two subject groups in each experiment are 30 and 70

We observe that in setting 1, where there exists no hidden variable or confounder, all methods, including our proposed method, have reasonably small FDR values. But in setting 2, where there exists a hidden effect of a confounder, the Fisher’s and weighted Z-score methods without any empirical adjustments perform very poorly with extremely high FDR values. In the presence of a hidden variable which does not act as confounder in setting 3, all methods performed similarly, except EAMA which had slightly higher FDR (0.11) compared to the other methods. Figure 1 shows the sensitivity, specificity, and FNR values of our proposed method and all the other methods in comparison, for the three simulation settings. We observed that all the methods have very similar sensitivity and FNR values in all settings. The specificity values of all methods, except the Fisher’s and weighted Z-score methods without any empirical adjustments, are also similar in all settings. The Fisher’s and weighted Z-score methods have low specificity values in setting 2 in the presence of hidden confounder.

#### Varying sample sizes of experiments

In this simulation scenario, we considered varying sample sizes of the experiments. We considered *N*_*i*_ = *N*_*i* − 1_ + 10, *i* = 2, …, *K* and *N*_1_ = 80. The subjects were equally divided between the two groups. Table 2 shows the FDR values for the three simulation settings under this scenario.

**Table 2:** FDR of our proposed meta-analysis method (BACON adjusted Weighted Z) and the other methods in comparison with unequal sample sizes of the experiments

Setting	Method	FDR
1	Fisher	0.05
	EAMA	0.06
	BACON adjusted Fisher	0.05
	weighted Z	0.05
	EB adjusted weighted Z	0.03
	BACON adjusted weighted Z	0.05
2	Fisher	0.89
	EAMA	0.05
	BACON adjusted Fisher	0.03
	weighted Z	0.90
	EB adjusted weighted Z	0.06
	BACON adjusted weighted Z	0.04
3	Fisher	0.05
	EAMA	0.12
	BACON adjusted Fisher	0.05
	weighted Z	0.05
	EB adjusted weighted Z	0.03
	BACON adjusted weighted Z	0.05

The results were very similar to what we observed before with varying sample sizes in the subject groups for all methods in all three settings, where the Fisher’s method and weighted Z-score method, without empirical adjustments, had very high FDR values in setting 2, and the FDR of EAMA was slightly high (0.12) in presence of a hidden variable which does not act as confounder. The sensitivity, and FNR values were similar for all methods in all settings, while the specificity values of the Fisher’s method and weighted Z-score method, without empirical adjustments, were very low in setting 2 (supplementary Fig. 1).

Since in many biological experiments the sample sizes are lower, we reduced the sample sizes of the experiments and compared the performances of the methods. We considered *N*_*i*_ = *N*_*i* − 1_ + 6, *i* = 2, …, *K* and *N*_1_ = 20. The FDR values for the three settings are shown in Table 3.

**Table 3:** FDR of our proposed meta-analysis method (BACON adjusted Weighted Z) and the other methods in comparison with reduced and unequal sample sizes of the experiments

Setting	Method	FDR
1	Fisher	0.06
	EAMA	0.09
	BACON adjusted Fisher	0.06
	weighted Z	0.05
	EB adjusted weighted Z	0.02
	BACON adjusted weighted Z	0.05
2	Fisher	0.68
	EAMA	0.03
	BACON adjusted Fisher	0.03
	weighted Z	0.86
	EB adjusted weighted Z	0.13
	BACON adjusted weighted Z	0.05
3	Fisher	0.05
	EAMA	0.06
	BACON adjusted Fisher	0.05
	weighted Z	0.05
	EB adjusted weighted Z	0.06
	BACON adjusted weighted Z	0.05

In setting 1, the FDR values of all the methods, except EAMA, were similar, where EAMA tends to have slightly high value (0.09). In setting 2, the performances of the Fisher’s method and the weighted Z-score method, without empirical adjustments, were consistently poor in the presence of hidden confounder. Additionally, the performance of the EB-adjusted weighted Z-score method gets worse with higher FDR (0.13). In setting 3, the FDR values of all the methods were similar. The sensitivity, specificity, and FNR values of all methods were very similar to what we observed before (supplementary Fig. 2).

Additionally, we considered a simulation scenario where different set of genes were differentially expressed across the experiments. We considered 500 genes as differentially expressed in the first five experiments and a separate set of 500 genes as differentially expressed in the remaining five experiments. This resulted in a total of 1000 genes as differentially expressed in at least one experiment. The sample sizes of the experiments were *N*_*i*_ = *N*_*i* − 1_ + 6, *i* = 2, …, *K* and *N*_1_ = 20. All the other choices of the parameters were same as before in all three settings. Supplementary table 1 shows the performances of all the methods in all three settings based on 500 Monte-Carlo iterations. We observed very similar performances of all the methods as we observed in the previous scenario with same genes as differentially expressed across experiments.

#### Reduced differential expression between the subject groups

In this scenario, we considered a reduced magnitude in differential expression of the genes between the two subject groups. To achieve this, the interaction terms were generated so that the absolute differences in the log expression values of the 1000 differentially expressed genes between the two groups was two and for all the remaining genes was zero. Additionally, we considered varying sample sizes of the experiments as we previously observed differences in performances of the methods under this scenario. We considered *N*_*i*_ = *N*_*i* − 1_ + 10, *i* = 2, …, *K* and *N*_1_ = 80. The results are shown in Table 4.

**Table 4:** Performances of our proposed meta-analysis method (BACON adjusted Weighted Z) and the other methods in comparison under reduced differential expressions between subject groups and varying experiment sample sizes

Setting	Method	Performance assessment measure			
		Sensitivity	Specificity	FDR	FNR
1	Fisher	1.00	0.99	0.05	0.00
	EAMA	1.00	0.99	0.09	0.00
	BACON adjusted Fisher	1.00	0.99	0.05	0.00
	weighted Z	1.00	0.99	0.05	0.00
	EB adjusted weighted Z	1.00	0.99	0.06	0.00
	BACON adjusted weighted Z	1.00	1.00	0.05	0.00
2	Fisher	0.44	0.13	0.95	0.34
	EAMA	0.40	1.00	0.003	0.34
	BACON adjusted Fisher	0.40	1.00	0.02	0.06
	weighted Z	0.55	0.01	0.94	0.84
	EB adjusted weighted Z	1.00	1.00	0.04	0.00
	BACON adjusted weighted Z	1.00	1.00	0.02	0.00
3	Fisher	0.99	1.00	0.05	0.00
	EAMA	0.99	1.00	0.02	0.00
	BACON adjusted Fisher	0.99	1.00	0.02	0.00
	weighted Z	1.00	1.00	0.05	0.00
	EB adjusted weighted Z	1.00	1.00	0.03	0.00
	BACON adjusted weighted Z	1.00	1.00	0.02	0.00

In setting 1, all methods perform well, except EAMA, which had slightly high FDR (0.09). Both EB adjusted weighted Z and our proposed method performed similar, however, the former had a slightly high FDR (0.06) in setting 1. In setting 2, where there exists an effect of hidden confounder, huge differences in the performances can be observed. Specifically, Fisher’s method and the weighted Z-score method without any empirical adjustments had very poor performances with low sensitivity and specificity values, and high FDR and FNR values. EAMA had low sensitivity and high FNR values, and the BACON-adjusted Fisher method had low sensitivity value. But both EB-adjusted weighted Z and our proposed method performed similarly. In setting 3, in presence of hidden variable which does not act as confounder, all methods had very similar performances.

Overall, summarizing from all the simulation results, we find that our proposed BACON adjusted weighted Z-score method has been the most consistent in maintaining the high levels of sensitivity and specificity while maintaining low or acceptable levels of false positive and false negative. Although EB-adjusted weighted Z is a good competitor of BACON adjusted weighted Z in terms of sensitivity, specificity, and FNR values, there exist instances in presence of hidden confounder (Table 3, and supplementary table 1) where EB-adjusted weighted Z has unacceptable FDR values that are much higher than the nominal type-I error rate.

### Lung cancer data

We considered five lung cancer gene expression datasets, namely Bhattacharjee [23], GSE11969 [24], GSE29016 [25], GSE30219 [26], and GSE43580 [27]. These datasets were previously normalized and processed by Hughey JJ et al. [28] which are available at [29]. Each dataset had normalized gene expression levels for 7200 genes for participants with different types of lung cancer. We aimed to identify the set of differentially expressed genes between the participants with Adenocarcinoma (AD) and Squamous cell carcinoma (SQ). Four of the datasets (GSE11969, GSE29016, GSE30219, and GSE43580) had information on the smoking status, gender, and age of the participants. All participants with missing covariates were removed from the analysis. Table 5 shows the characteristics of the participants for the two cancer types in each dataset.

**Table 5:** Characteristics of the Adenocarcinoma (AD) and Squamous cell carcinoma (SQ) participants in each of the five lung cancer datasets

Dataset	Cancer type N (%)		Smoking status N (%)			Gender N (%)		Age years (mean ± SD)
	AD	SQ	Never	Former	Current	Female	Male	
Bhattacharjee (*N* = 81)	60 (74.1)	21 (25.9)	–	–	–	–	–	–
GSE11969 (*N* = 125)	90 (72.0)	35 (28.0)	46 (36.8)	–	79 (63.2)	45 (36.0)	80 (64.0)	62.3 ± 9.6
GSE29016 (*N* = 47)	36 (76.6)	11 (23.4)	10 (21.3)	–	37 (78.7)	23 (48.9)	24 (51.1)	67.3 ±11.0
GSE30219 (*N* = 145)	84 (57.9)	61 (42.1)	10 (6.9)	68 (46.9)	67 (46.2)	24 (16.6)	121 (83.4)	62.3 ± 9.1
GSE43580 (*N* = 144)	72 (50.0)	72 (50.0)	28 (19.4)	20 (13.9)	96 (66.7)	27 (18.8)	117 (81.2)	59.5 ± 9.0

We tested for differential expression of the genes between AD and SQ participants using the ‘limma’ package in Bioconductor [22], adjusting for the available covariates, for each dataset separately. The raw *p*-values and the direction of the effects of the genes were stored for the meta-analysis. We applied our proposed meta-analysis method to identify the set of differentially expressed genes between AD and SQ lung cancer participants. The empirically estimated null distribution of the z-scores, using BACON [19], had mean − 0.34 and standard deviation (SD) 1.91. This suggests that the empirically estimated null distribution of the z-scores is much deviated from the theoretical null distribution, *N*(0, 1). After multiple testing correction with BH method [20], we identified 2957 differentially expressed genes between AD and SQ participants at 5% significance level.

**Fig. 2:**
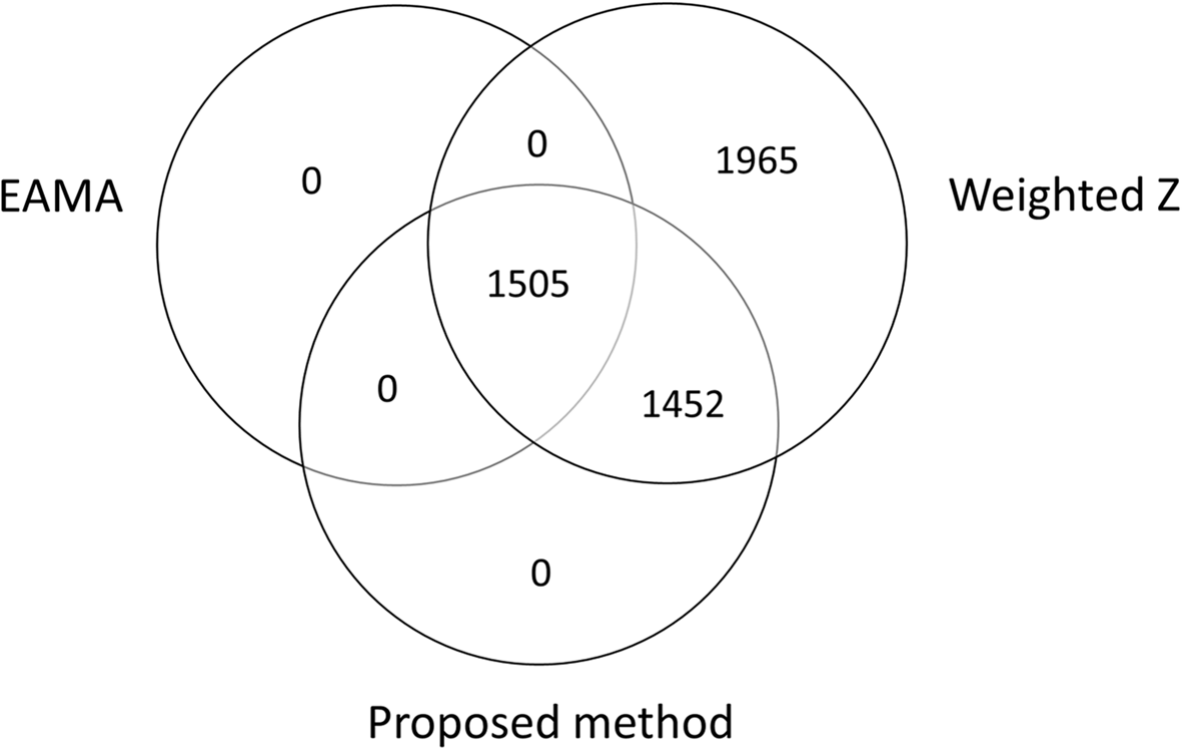
Venn diagram showing the number of differentially expressed genes identified by the meta-analysis methods. This figure shows the overlap between the number of differentially expressed genes, significant at BH-adjusted *p*-value cutoff of 0.05, identified by the proposed method, the naïve weighted Z-score method and EAMA

For comparison, we also applied the naïve weighted Z-score method as well as our previously proposed method, EAMA, to identify the set of differentially expressed genes. The naïve weighted Z-score method identified 4922 differentially expressed genes, while EAMA identified 1505 differentially expressed genes, at BH adjusted *p*-value cutoff of 0.05. A Venn diagram showing the overlap of the number of differentially expressed genes identified by our proposed method with the other two methods in comparison is given in Fig. 2. All the genes identified by EAMA were also identified by both our proposed method and the naïve weighted Z-score method. Additionally, all the genes identified by the proposed method were also identified by the naïve weighted Z-score method. Identification of so many differentially expressed genes by the naïve weighted Z-score method indicates possibility of high gross false discoveries. EAMA identified much lesser number of genes compared to our proposed method at the same BH adjusted *p*-value cutoff, which might reflect a situation where EAMA has lower sensitivity and/or high non false discovery rate, similar to setting 2 in Table 4. We also checked the performances of the methods without adjusting for the covariates in the studies, assuming they are hidden. The pattern of performances of the methods were very similar to what we observed after adjusting for the observed covariates, where the naïve weighted Z-score method identified a large number of differentially expressed genes and EAMA identified much lesser number of genes compared to our proposed method. Note that, even after adjusting for the observed covariates, there still might exist potential hidden confounders underlying these studies which remained unaccounted for in all our analyses. We proceed with the results adjusting for the covariates with the aim to account for all possible covariates effects that have been observed.

In order to identify biological pathways associated with the significant list of genes identified by all three methods, we performed functional annotation analysis using the software, called DAVID [30, 31]. Some of the top pathways overrepresented in the significant list of genes include cell cycle, DNA replication, pathways in cancer, and p53 signaling pathway, which has been frequently found to be associated with lung cancer [32–34]. We also identified the pathways overrepresented in the significant list of genes identified only by our proposed method. Pathways related to lung cancer, such as non-small cell lung cancer and Foxo signaling pathway [35], were significantly overrepresented in our gene list.

## Discussion

Meta-analysis is a popular tool for combining hypothesis testing results from multiple studies. It is extensively used in genomic studies, clinical studies, psychological studies, and other social sciences applications. The field of genomic experiments have undergone major changes in the past few years with the advent of modern high-throughput technologies. One such change is that thousands of genes can be analyzed simultaneously nowadays which, in turn, leads to simultaneous testing of thousands of hypotheses. While combining such large-scale multiple hypotheses testing results from multiple studies, the traditional meta-analysis approaches involving *p*-value combinations fail to make use of the large-scale aspect of the data. For instance, large-scale hypotheses testing allows empirical estimation of the parameters of null distributions without having to rely on some theoretically set null parameters. However, such provisions of empirically adjusting the null distributions are not accommodated by the classical *p*-value combination methods. A possible consequence of relying only on theoretical null distributions can be gross false discoveries and inaccurate inference from meta-analysis. As discussed in this article, this problem becomes more profound whenever there is a possibility of presence of some unobserved variables or unmeasured confounders. In this article we discussed some recent developments in estimating empirical null distributions and proposed ways for incorporating such empirical null distributions in meta-analysis of large-scale genomic experiments. Finally, we proposed an empirically adjusted weighted *p*-value combination approach which estimates the empirical null distribution parameters through a Bayesian framework. We demonstrated its robustness and superiority over other meta-analysis approaches through a wide variety of simulation settings that mimic large-scale genomic testing experiments. We also applied our proposed method in meta-analysis of multiple lung cancer gene-expression studies to obtain biologically meaningful results. Although we mostly focused on microarray studies in this article, our proposed method can be easily applied for meta-analysis of expression data from other platforms (e.g., next-generation sequencing) or other type of genomic studies (e.g., DNA methylation, SNP data) as long as one can obtain *p*-values for each genomic feature from multiple studies.

The proposed method assumes a common null distribution across all studies, which is estimated empirically instead of relying on a theoretical null distribution. There exist meta-analysis methods that do not necessarily assume a common null distribution to account for between studies variability. However, such methods are primarily model-based approaches, e.g., random effects model, which is a different category of meta-analysis that requires information on the individual effect sizes and their corresponding standard errors to obtain a measure of between-study variability [36]. In many situations, the individual effect size estimates and the corresponding standard errors are not available. Therefore, in this article, we have focused on those meta-analysis methods that require only *p*-values from individual studies.

In this article we aimed at improving the meta-analysis method of large-scale genomic testing studies by modifying the classical *p*-value combination methods through empirical adjustments. These classical p-value combination methods aim to test for significance of a gene in at least one of the component studies and the method proposed in this article is based on the same principle of significance testing. There exists another approach of meta-analysis of significance testing results that focuses on testing for significance of a gene in the majority (e.g., 70%) of the component studies. There have been some recent *p*-value combination methods that aimed for this second type of meta-analysis [11, 37]. Since these methods test hypotheses which are conceptually different from the hypotheses we are testing and have a different aim, we have not discussed them in this article. Nevertheless, the empirical adjustments, which we applied in our meta-analysis method, can also be extended to the meta-analysis methods of the second type if the main aim is to find significant genes in the majority of component studies. In future, we plan to pursue this approach of empirical adjustments to the second type of meta-analysis.

## Conclusion

In this article, we have highlighted the drawbacks of the classical *p*-value combination methods for significance testing in large-scale genomic experiments. These classical *p*-value combination methods rely on a theoretical null distribution which can be different from the true null distribution especially in the presence of confounding variables in large observational studies. We have proposed a robust meta-analysis approach of *p*-value combination which modifies the *p*-values through the computation of an empirical null distribution. Our proposed meta-analysis approach can account for the effects of unobserved variables and confounders and has been shown to perform better than the classical *p*-value combination methods and other competing meta-analysis techniques. Overall, we believe that our proposed meta-analysis approach can help in accurate identification of truly significant genes by combining the findings of multiple large-scale genomic experiments.

## Supplementary Information

**Additional file 1: Supplementary table 1.** Performances of our proposed meta-analysis method and the other methods in comparison when the set of differentially expressed genes vary between experiments. The sample sizes of the experiments are *N*_*i*_ = *N*_*i* − 1_ + 6, *i* = 2, …, 10 and *N*_1_ = 20. **Supplementary figure 1.** Performances of the meta-analysis methods with unequal sample sizes of the experiments. **Supplementary figure 2.** Performances of the meta-analysis methods with reduced and unequal sample sizes of the experiments.

**Additional file 2. **R code for the proposed method (BACON adjusted Weighted Z).

## Data Availability

The datasets generated and/or analyzed during the current study are available at https://zenodo.org/record/16006 or upon request from the author(s).

## References

[1] Karim JN, Bradburn E, Roberts N, Papageorghiou AT, ACCEPTS study. First trimester ultrasound for the detection of fetal heart anomalies: a systematic review and meta-analysis. Ultrasound Obstet Gynecol. 2021. 10.1002/uog.23740. 10.1002/uog.23740 PMC9305869 34369613

[2] Reese SE, Xu CJ, den Dekker HT, Lee MK, Sikdar S, Ruiz-Arenas C (2019). Epigenome-wide meta-analysis of DNA methylation and childhood asthma. J Allergy Clin Immunol.

[3] Kröger W, Mapiye D, Entfellner JD, Tiffin N (2016). A meta-analysis of public microarray data identifies gene regulatory pathways deregulated in peripheral blood mononuclear cells from individuals with systemic lupus erythematosus compared to those without. BMC Med Genet.

[4] Panagiotou OA, Willer CJ, Hirschhorn JN, Ioannidis JPA (2013). The power of meta-analysis in genome-wide association studies. Annu Rev Genomics Hum Genet.

[5] Evangelou E, Maraganore DM, Ioannidis JPA (2007). Meta-analysis in genome-wide association datasets: strategies and application in Parkinson disease. PLoS One.

[6] Bhattacharjee S, Rajaraman P, Jacobs KB, Wheeler WA, Melin BS, Hartge P (2012). A subset-based approach improves power and interpretation for the combined analysis of genetic association studies of heterogeneous traits. Am J Hum Genet.

[7] Lee S, Teslovich TM, Boehnke M, Lin X (2013). General framework for meta-analysis of rare variants in sequencing association studies. Am J Hum Genet.

[8] Fromer M, Roussos P, Sieberts SK, Johnson JS, Kavanagh DH, Perumal TM (2016). Gene expression elucidates functional impact of polygenic risk for schizophrenia. Nat Neurosci.

[9] Bock C (2012). Analysing and interpreting DNA methylation data. Nat Rev Genet.

[10] Rheinbay E, Nielsen MM, Abascal F, Wala JA, Shapira O, Tiao G (2020). Analyses of non-coding somatic drivers in 2,658 cancer whole genomes. Nature.

[11] Li Y, Ghosh D (2014). Meta-analysis based on weighted ordered P-values for genomic data with heterogeneity. BMC Bioinformatics.

[12] Fisher RA (1932). Statistical methods for research workers.

[13] Stouffer SA, Suchman EA, Devinney LC, Star SA, Williams RM, JR. (1949). The American soldier: adjustment during army life.

[14] Willer CJ, Li Y, Abecasis GR (2010). METAL: fast and efficient meta-analysis of genomewide association scans. Bioinformatics.

[15] Efron B (2004). Large-scale simultaneous hypothesis testing: the choice of a null hypothesis. J Am Stat Assoc.

[16] Leek JT, Scharpf RB, Bravo HC, Simcha D, Langmead B, Johnson WE (2010). Tackling the widespread and critical impact of batch effects in high-throughput data. Nat Rev Genet.

[17] Sikdar S, Datta S, Datta S (2017). EAMA: empirically adjusted meta-analysis for large-scale simultaneous hypothesis testing in genomic experiments. PLoS One.

[18] You C, Wu S, Zheng SC, Zhu T, Jing H, Flagg K (2020). A cell-type deconvolution meta-analysis of whole blood EWAS reveals lineage-specific smoking-associated DNA methylation changes. Nat Commun.

[19] van Iterson M, van Zwet EW, Heijmans BT, BIOS Consortium (2017). Controlling bias and inflation in epigenome- and transcriptome-wide association studies using the empirical null distribution. Genome Biol.

[20] Benjamini Y, Hochberg Y (1995). Controlling the false discovery rate: a practical and powerful approach to multiple testing. J R Stat Soc Ser B Methodol.

[21] Efron B (2007). Size, power and false discovery rates. Ann Stat.

[22] Ritchie ME, Phipson B, Wu D, Hu Y, Law CW, Shi W (2015). limma powers differential expression analyses for RNA-sequencing and microarray studies. Nucleic Acids Res.

[23] Bhattacharjee A, Richards WG, Staunton J, Li C, Monti S, Vasa P (2001). Classification of human lung carcinomas by mRNA expression profiling reveals distinct adenocarcinoma subclasses. Proc Natl Acad Sci.

[24] Takeuchi T, Tomida S, Yatabe Y, Kosaka T, Osada H, Yanagisawa K (2006). Expression profile–defined classification of lung adenocarcinoma shows close relationship with underlying major genetic changes and clinicopathologic behaviors. J Clin Oncol.

[25] Staaf J, Jönsson G, Jönsson M, Karlsson A, Isaksson S, Salomonsson A (2012). Relation between smoking history and gene expression profiles in lung adenocarcinomas. BMC Med Genet.

[26] Rousseaux S, Debernardi A, Jacquiau B, Vitte AL, Vesin A, Nagy-Mignotte H (2013). Ectopic activation of germline and placental genes identifies aggressive metastasis-prone lung cancers. Sci Transl Med.

[27] Tarca AL, Lauria M, Unger M, Bilal E, Boue S, Dey KK (2013). Strengths and limitations of microarray-based phenotype prediction: lessons learned from the IMPROVER Diagnostic Signature Challenge. Bioinformatics.

[28] Hughey JJ, Butte AJ (2015). Robust meta-analysis of gene expression using the elastic net. Nucleic Acids Res.

[29] The lung cancer datasets. https://zenodo.org/record/16006. Accessed 5 Dec 2020.

[30] Huang DW, Sherman BT, Lempicki RA (2009). Systematic and integrative analysis of large gene lists using DAVID bioinformatics resources. Nat Protoc.

[31] Huang DW, Sherman BT, Lempicki RA (2009). Bioinformatics enrichment tools: paths toward the comprehensive functional analysis of large gene lists. Nucleic Acids Res.

[32] Vincenzi B, Schiavon G, Silletta M, Santini D, Perrone G, Di Marino M (2006). Cell cycle alterations and lung cancer. Histol Histopathol.

[33] Xie M, Park D, Sica GL, Deng X (2020). Bcl2-induced DNA replication stress promotes lung carcinogenesis in response to space radiation. Carcinogenesis.

[34] Robles AI, Linke SP, Harris CC (2002). The p53 network in lung carcinogenesis. Oncogene.

[35] Maekawa T, Maniwa Y, Doi T, Nishio W, Yoshimura M, Ohbayashi C (2009). Expression and localization of FOXO1 in non-small cell lung cancer. Oncol Rep.

[36] Han B, Eskin E (2011). Random-effects model aimed at discovering associations in meta-analysis of genome-wide association studies. Am J Hum Genet.

[37] Song C, Tseng GC (2014). Hypothesis setting and order statistic for robust genomic meta-analysis. Ann Appl Stat.

