# Supplementary material for: Robust meta-analysis for large-scale genomic experiments based on an empirical approach
Source: BMC Med Res Methodol. 2022 Feb 10;22:43. doi: 10.1186/s12874-022-01530-y (PMC8832678; doi:10.1186/s12874-022-01530-y)

Supplementary table 1. Performances of our proposed meta-analysis method and the other methods in comparison when the set of differentially expressed genes vary between experiments. The sample sizes of the experiments are $N_{i}=N_{i-1}+6$, $i=2,\ldots,10$ and $N_{1}=20$.

| Setting | Method | Performance assessment measure | | | |
| --- | --- | --- | --- | --- | --- |
|  |  | Sensitivity | Specificity | FDR | FNR |
| 1 | Fisher | 1.00 | 0.99 | 0.06 | 0.00 |
|  | EAMA | 1.00 | 0.99 | 0.08 | 0.00 |
|  | BACON adjusted Fisher | 1.00 | 0.99 | 0.06 | 0.00 |
|  | weighted Z | 1.00 | 0.99 | 0.05 | 0.00 |
|  | EB adjusted weighted Z | 1.00 | 1.00 | 0.03 | 0.00 |
|  | BACON adjusted weighted Z | 1.00 | 0.99 | 0.05 | 0.00 |
| 2 | Fisher | 1.00 | 0.77 | 0.68 | 0.00 |
|  | EAMA | 0.99 | 1.00 | 0.02 | 0.00 |
|  | BACON adjusted Fisher | 0.99 | 1.00 | 0.03 | 0.00 |
|  | weighted Z | 0.96 | 0.31 | 0.87 | 0.02 |
|  | EB adjusted weighted Z | 1.00 | 0.99 | 0.11 | 0.00 |
|  | BACON adjusted weighted Z | 0.99 | 1.00 | 0.05 | 0.00 |
| 3 | Fisher | 1.00 | 0.99 | 0.05 | 0.00 |
|  | EAMA | 1.00 | 1.00 | 0.04 | 0.00 |
|  | BACON adjusted Fisher | 1.00 | 0.99 | 0.05 | 0.00 |
|  | weighted Z | 1.00 | 1.00 | 0.05 | 0.00 |
|  | EB adjusted weighted Z | 1.00 | 0.99 | 0.07 | 0.00 |
|  | BACON adjusted weighted Z | 1.00 | 1.00 | 0.05 | 0.00 |

Supplementary figure 1. Performances of the meta-analysis methods with unequal sample sizes of the experiments.

This figure shows the average sensitivity, specificity, and FNR values over 500 independent Monte-Carlo iterations of the proposed method and all the other methods in comparison. Results are shown for all three simulation settings. The sample sizes of the experiments are $N_{i}=N_{i-1}+10$, $i=2,\ldots,10$ and $N_{1}=80$.


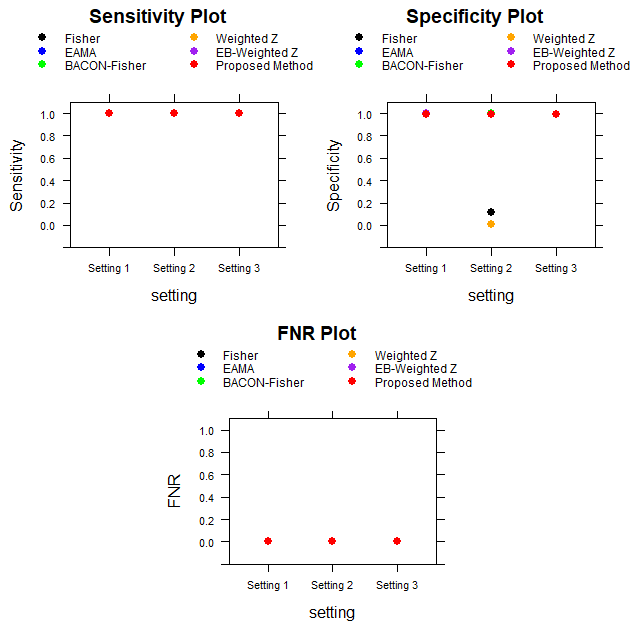


Supplementary figure 2. Performances of the meta-analysis methods with reduced and unequal sample sizes of the experiments.

This figure shows the average sensitivity, specificity, and FNR values over 500 independent Monte-Carlo iterations of the proposed method and all the other methods in comparison. Results are shown for all three simulation settings. The sample sizes of the experiments are $N_{i}=N_{i-1}+6$, $i=2,\ldots,10$ and $N_{1}=20$.


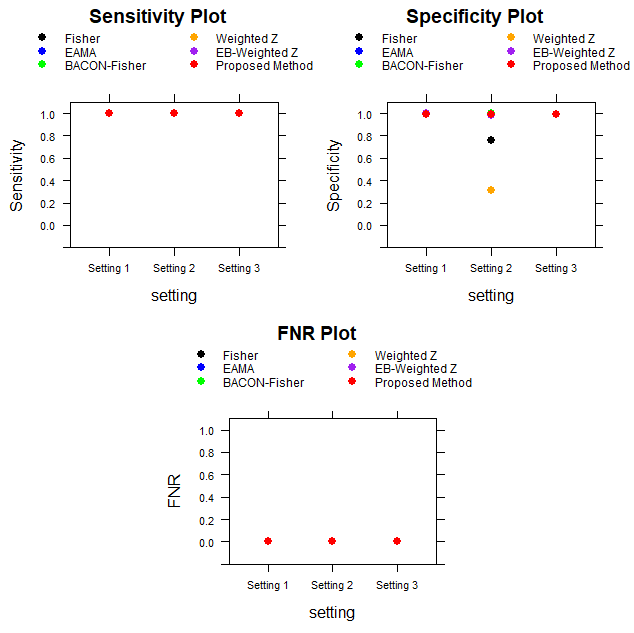

Supplement: Supplementary file 1 — Additional file 1: Supplementary table 1. Performances of our proposed meta-analysis method and the other methods in comparison when the set of differentially expressed genes vary between experiments. The sample sizes of the experiments are Ni = Ni − 1 + 6, i = 2, …, 10 and N1 = 20. Supplementary figure 1. Performances of the meta-analysis methods with unequal sample sizes of the experiments. Supplementary figure 2. Performances of the meta-analysis methods with reduced and unequal sample sizes of the experiments. [file 12874_2022_1530_MOESM1_ESM.docx]
